# The Impact of Gross Motor Function on the Oral Health-Related Quality of Life in Young Adults with Cerebral Palsy in Saudi Arabia

**DOI:** 10.1155/2020/4590509

**Published:** 2020-02-28

**Authors:** Sharat Chandra Pani, Saja Fahad AlEidan, Rawan Nasser AlMutairi, AlJawharah Ali AlAbsi, Dalal Nasser AlMuhaidib, Hessa Faisal AlSulaiman, Najd Waleed AlFraih

**Affiliations:** ^1^Schulich School of Dentistry and Pharmacy, Western University, London, ON, Canada; ^2^College of Dentistry, Riyadh Elm University, Riyadh, Saudi Arabia

## Abstract

**Background:**

There is evidence that gross motor function impacts the health-related quality of life of young adults with cerebral palsy. This study aimed to assess gross motor function, oral health and oral health-related quality of life (OHRQoL), and the relationship between them in young adults with cerebral palsy.

**Methods:**

The sample comprised 46 individuals aged between 13 and 17 years with Gross Motor Function Classification Scores (GMFCS) ranging from level I to level III. The individuals and their parents were administered an Arabic version of the child perception questionnaire for adolescents. Parental and child perception scores, DMFT, and gingival index were compared across GMFCS levels using the one-way ANOVA and Scheffe's post hoc test.

**Results:**

Children with level III GMFCS had a significantly higher child perception score (CPQ) and parental perception score (PPQ) than those with level I or level II scores. There was a significant association between function (GMFCS) and the CPQ score in children (*p* = 0.016). No significant associations were found between the CPQ score and either dental caries (DMFT) or gingival bleeding (GI) scores. Children with GMFCS level III had a significantly higher DMFT (*p* = 0.016). No significant associations were found between the CPQ score and either dental caries (DMFT) or gingival bleeding (GI) scores. Children with GMFCS level III had a significantly higher DMFT (*p* = 0.016). No significant associations were found between the CPQ score and either dental caries (DMFT) or gingival bleeding (GI) scores. Children with GMFCS level III had a significantly higher DMFT (

**Conclusion:**

Motor function has a significant impact on both the oral health and the OHRQoL of adolescents and young adults with spastic cerebral palsy.

## 1. Introduction

Cerebral palsy (CP) is not a specific disease entity but rather a collection of disabling disorders caused by insult and permanent damage to the brain in the prenatal and perinatal periods, during which time the central nervous system is still maturing [1]. This disability might involve muscle weakness, stiffness or paralysis, poor balance or irregular gait, and uncoordinated or involuntary movements [1]. It has been reported that cerebral palsy was one of the most common neurological disorders among Saudi children with a prevalence of 42 per 10000 live births (0.42%) [2].

The health-related quality of life (HRQoL) of adolescents and young adults with cerebral palsy has recently received attention in literature [3, 4]. Researchers have pointed out that the level of functional activity of the individual with CP plays an important role in the overall perception of their HRQoL [5–7]. With growing emphasis on the inclusion of individuals with CP into mainstream activities, it has been pointed out that it is important to be able to measure both function [8, 9] and HRQoL of these individuals [3].

Oral health-related quality of life (OHRQoL) is a multidimensional construct that includes a subjective evaluation of the individual's oral health, functional well-being, emotional well-being, expectations and satisfaction with care, and sense of self. It has wide-reaching applications in survey and clinical research [10–13]. The OHRQoL is an integral part of general health and well-being. There have been several scales specifically designed to quantitatively assess the OHRQoL in children and young adults [10,13–16]. The CPQ11-14 has proven to be a good predictor of quality of life among adolescents and young adults [7].

The OHRQoL of individuals with CP has only recently been receiving attention in literature with studies looking into the OHRQoL of both children with CP and their caregivers [17,18]. The traditional method of studying the OHRQoL in children with CP has focused on the experiences of the parents [17,19]. There is, however, some evidence to suggest that significant variations exist in the perception of OHRQoL reported by parents when compared to the values reported by their children with CP [20].

Function plays an important role in the overall health-related quality of life of individuals with cerebral palsy [20–22]. There are several methods of evaluating motor function in children with cerebral palsy of which the gross motor function classification system (GMFCS) is widely accepted. The revised version of the GMFCS has been found to be an acceptable tool for assessment of motor function in adolescents [7]. While there is growing evidence on the role of function on HRQoL of individuals with CP, there is little known about the role it plays on the OHRQoL. Given the relationship between function and physical development, the age study of function in young adults necessitates the examination of a relatively narrow age range of individuals.

This study aimed to assess gross motor function, oral health and oral health-related quality of life (OHRQoL), and the relationship between them in young adults with CP aged between 13 and 17 years attending a rehabilitation center in Riyadh, Saudi Arabia.

## 2. Materials and Methods

### 2.1. Ethical Approval

The study was registered with the research center of the Riyadh Elm University (FUGRP/2018/140), and ethical approval was obtained from the Institutional Review Board of the Institution (RC/IRB/2018/1054). Ethical approval was also obtained from the ethical committee of the Prince Sultan Humanitarian City. Written informed consent was obtained from all parents and participants in the study, and assent was obtained from each participant before the administration of the questionnaire and oral examination.

### 2.2. Study Design

A cross-sectional study design was used to measure the OHRQoL in individuals with CP aged between 13 and 17 years and their parents.

### 2.3. Recording of Variables

A validated Arabic Version of the Parental Perception Questionnaire and Child Perception Questionnaire was used for the determination of OHRQoL [15]. The questionnaire comprised a parental component that was filled in by the parent and a child component that was filled in by the examiner after speaking to the individual with cerebral palsy. Each component comprised 16 questions divided into four domains: oral symptoms, functional limitations, emotional, and social well-being. In addition, the questionnaire comprises an 8-question family impact score that is answered by the parent [23–25]. The total number of decayed, missing, and filled permanent teeth (DMFT) and the gingival index were recorded to assess caries levels and gingival status, respectively. The teeth were examined using WHO category II criteria: clinical examination with dental unit light and without radiographs. Examiners were calibrated for the use of the recording form and for the detection of dental caries. A total of three examiners examined the patients (NaF, SaE, and AaA). The overall kappa for the total number of decayed, missing, and filled was 0.871 among three examiners. No kappa measurements were taken for gingival bleeding scores. However, as recommended by the WHO, examiners were trained for the measurement of gingival problems.

### 2.4. Sample Selection and Administration of Questionnaire

The sample comprised patients who were attending the outpatient clinics of the Prince Sultan Humanitarian City, Riyadh. The patients were aged between 13 and 17 years and were diagnosed with mild to moderate cerebral palsy and had been classified by the pediatric neurologist at the center as being either level I, level II, or level III on the Gross Motor Function Classification System (GMFCS). All participants had to be able to walk independently (at least with a waking aid). Only children with spastic monoplegia, hemiplegia or spastic diplegia were included in the study. Children with visual or hearing impairments, those with other forms of CP, and those with any additional disease were excluded from the sample.

### 2.5. Statistical Analyses

The one-way ANOVA and Scheffe's post hoc test were used to compare OHRQoL (CPQ or PPQ), dental caries (DMFT), and gingival status (GI) scores among children with different levels of function (GMFCS score). All statistical analyses were performed using IBM-SPSS v25 data processing software (IBM Corp. Armonk NY, USA), and the alpha for all tests was set at *p* < 0.05.

## 3. Results

The sample comprised 45 adolescents (21 male 25 female) with cerebral palsy reporting for outpatient dental care at the Prince Sultan Humanitarian City. The adolescents ranged between 13 and 17 years of age (mean age 15.2, SD±2.9 years). Diplegia was the most common form of limb involvement seen followed by monoplegia and hemiplegia (Table 1). When the CP and level of movement of the participants were compared, it was observed that there were fewer children with hemiplegia (*n* = 9) than with diplegia (*n* = 19) or monoplegia (*n* = 18). It was also observed that a majority of the sample population (*n* = 31) were on some forms of long-term medication. Almost all the children in the sample (*n* = 42) had a history of surgery (Table 1).

Most of the primary guardians were mothers (*n* = 35), while fathers (*n* = 7) and caregivers (*n* = 4) were less frequent as the primary caregiver. The demographic profile of the families interviewed was a mixture of the different sociodemographic groups (Table 2). When oral health-related quality of life and parental perception of OHRQoL were examined, it was observed that there was a significant increase in child perception scores with a decrease in the level of function. The same was true for parental perception scores (Table 3). Scheffe's post hoc test showed that there was a significant difference between children with level I and level III GMFCS scores (*p* < 0.05). Scheffe's post hoc test showed that there were no significant differences in the child perception scores between children with level I and level II GMFCS score (*p*=0.171) or between children with level II and level III GMFCS scores (*p*=0.092). When parental perceptions scores were compared, it was observed that there was no significant difference between parents of children with level I or level II GMFCS scores (*p*=0.566). However, parents of children with GMFCS score III showed a significantly higher score on the parental perception questionnaire (*p* < 0.05).

When the oral health of children was compared among children with different levels of function, it was observed that the one-way ANOVA showed that there were significant differences in both DMFT and GI scores. The post hoc tests revealed that there were no significant differences in DMFT between individuals with level I and level II function (*p*=0.670), but individuals with level III functional impairment had a significantly higher DMFT score (*p* < 0.05). When the GI scores were compared, it was observed that individuals with level II had significantly higher sores than those with level I function (*p* < 0.05) but significantly lower GI scores than those with level III function (*p* < 0.05) (Table 4).

## 4. Discussion

The oral health of children and young adults with cerebral palsy has been studied since 1970s. However, it is only recently that studies have sought to look at the impact of that impaired oral health on the quality of life of these individuals [15,17,19]. This study sought to assess the impact of overall motor function and oral health on the oral health-related quality of life of individuals with cerebral palsy in Saudi Arabia.

Saudi Arabia has a documented history of high dental caries in children and adolescents, even in the absence of underlying systemic disease [23]. There is also documented evidence that children and adolescents in Saudi Arabia have poor oral hygiene practices [24]. It was for this reason that the study chose not to include a control group of CP-free children and adolescents' age and gender matched to the population.

The role of motor function in the overall health-related quality of life has received considerable attention in recent literature [3, 25, 26]. The GMFCS is a validated tool that has been used repeatedly as a measure of motor function in individuals with cerebral palsy [19]. The decision to limit the inclusion criteria in the present study up to level III GMFCS was based on the fact that previous studies have shown individuals with GMFCS level IV and level V to have a compromised motor function that would require assistance [6, 19, 25].

Studies have also shown a negative correlation between the GMFCS score and the overall activity participation [3,5,27]. The fact that the results of our study found a similar relationship with CPQ scores, where there was a worse OHRQoL with increasing GMFCS level, seems to highlight the role function in oral health. This finding is further substantiated by the trend of higher dental caries and gingival index scores with a progression in the GMFCS level.

The literature on the role of motor function on oral health of young adults with cerebral palsy is scant. One of the reasons for this is, perhaps, the fact that very often, a decreased motor function is accompanied by the use of a caregiver to provide oral health care [18]. It was for this reason that the sample in this study was restricted to individuals with GCMFS scores no greater than level III. Furthermore, this was also the reason for making sure that the participant being responsible for their own oral hygiene was an essential inclusion criterion.

There has been debate in the past over whether children and adolescents with cerebral palsy are effective rapporteurs of their quality of life [17,18]. There is ample evidence from medical literature to support that individuals with CP are effective rapporteurs of their own health-related quality of life [3, 4, 22, 26]. There is also evidence to suggest that while qualitative data from individuals can be useful, in most cases, the use of validated questionnaires in individuals with CP has found to be a simple and effective measure of quality of life [3]. The decision to allow children to report their overall OHRQoL was also because the study was limited to children without intellectual disability who were attending regular schools.

The impact of cerebral palsy on the family is the one that has been extensively studied [7, 14, 28]. In keeping with previous research on OHRQoL in children and adolescents with CP, the family impact scale was completed only by the parent [14, 18].

The results of the study need to be viewed considering certain limitations. The term cerebral palsy is an umbrella term used to classify different patterns of limited motor function. It was, therefore, necessary to restrict our study to only children with spastic cerebral palsy in keeping with the methodology used by similar studies assessing health-related quality of life. But, the limitation of this approach is that the findings may not be generalizable to all forms of CP. Further research with a larger sample is needed to confirm the generalize these findings to individuals with other forms of CP.

The study, however, has some important strength. The study reinforces the idea that function and activity participation are critical to the ability of young adults with cerebral palsy to lead independent and productive lives [3–5]. Clinically, the study also shows that young adults with CP are aware of their oral health challenges, and dentists in the 21^st^ century should be ready to meet these challenges.

## 5. Conclusions

With the limitations of the current study, we can conclude that motor function has a significant impact on both the oral health and the oral health-related quality of life of adolescents with spastic cerebral palsy. Further research on the topic is needed to prove the generalizability of these findings to all forms of cerebral palsy.

## Figures and Tables

**Table 1:** Description of the study population.

		Male		Female		Total	%
		*N*	%	*N*	%		
Limb	Monoplegia	10	47.6	8	32.0	18	39.1
Involvement	Hemiplegia	6	28.6	3	12.0	9	19.6
	Diplegia	5	23.8	14	56.0	19	41.3

Medication	No long-term medication	5	23.8	10	40.0	15	32.6
	Long-term medication	16	76.2	15	60.0	31	67.4

History of surgery	No	2	9.5	2	8.0	4	8.7
	Yes	19	91.5	23	92.0	42	91.3

GMFCS score	Level I	9	42.8	8	32.0	17	36.9
	Level II	7	33.4	7	28.0	14	30.5
	Level III	5	23.8	10	40.0	15	32.6

**Table 2:** Sociodemographic profile of the population.

		Count	Column *N* (%)
Monthly family income	<5000 SR	6	13.0
	5000SR–10000SR	13	28.3
	10000SR–20000SR	18	39.1
	20000–40000SR	7	15.2
	<40000SR	2	4.3

Education of father	Elementary school	13	28.3
	High school	18	39.1
	Graduate degree	13	28.3
	Postgraduate degree	2	4.3

Education of mother	Elementary school	13	28.3
	High school	19	41.3
	Graduate degree	14	30.4
	Postgraduate degree	0	0.0

Employment status of father	Unemployed	19	41.3
	Employed	27	58.7

Employment status of mother	Unemployed	35	76.1
	Employed	11	23.9

**Table 3:** Child and parental perception of OHRQoL according to the level of function.

		Mean	Std. deviation	95% confidence interval for mean		*F* ^*∗*^	Sig
				Lower bound	Upper bound		
CPQ	GMFCS level I^a^	35.0588	9.74981	30.0459	40.0717	9.065	0.001^*∗∗*^
	GMFCS level II^ab^	40.9286	8.63325	35.9439	45.9133		
	GMFCS level III^b^	47.8000	6.42762	44.2405	51.3595		

PPQ	GMFCS level I^a^	36.7647	9.37103	31.9466	41.5828	6.859	0.003^*∗∗*^
	GMFCS level II^a^	40.2143	8.14464	35.5117	44.9169		
	GMFCS level III^b^	48.2000	8.90586	43.2681	53.1319		

^*∗*^Calculated using one-way ANOVA. ^*∗∗*^Differences significant at *p* < 0.05. ^a,b^Differences in superscript indicate significant difference at *p* < 0.05 when compared using Scheffe's post hoc test.

**Table 4:** Impact of functional ability on oral health.

GMFCS		Mean	Std. deviation	95% confidence interval for mean		*F* ^*∗*^	Sig
				Lower bound	Upper bound		
DMFT	Level I^a^	1.1176	1.40900	0.3932	1.8421	7.342	0.002^*∗∗*^
	Level II^a^	1.7143	2.05421	0.5282	2.9004		
	Level III^b^	3.5333	2.03072	2.4088	4.6579		

GI	Level I^a^	4.7647	3.63197	2.8973	6.6321	8.218	0.001^*∗∗*^
	Level II^b^	7.2143	4.11710	4.8371	9.5914		
	Level III^c^	10.4000	4.06729	8.1476	12.6524		

^*∗*^Calculated using one-way ANOVA. ^*∗∗*^Differences significant at *p* < 0.05. ^a,b,c^Differences in superscript indicate significant difference at *p* < 0.05 when compared using Scheffe's post hoc test.

## Data Availability

The data and additional materials are made available from the corresponding author upon request.
